# Assessment of Maternal and Fetal Outcomes in Pregnancy Complicated by Fibroid Uterus

**DOI:** 10.7759/cureus.22052

**Published:** 2022-02-09

**Authors:** Upasana Pandit, Meenakshi Singh, Rajesh Ranjan

**Affiliations:** 1 Department of Obstetrics and Gynaecology, Hindalco Hospital, Renukoot, IND; 2 Department of Obstetrics and Gynaecology, Lady Hardinge Medical College, Delhi, IND; 3 Community Medicine, Noida International Institute of Medical Sciences, Gautam Buddha Nagar, IND

**Keywords:** uterus, primigravida, multigravida, labour pain, fibroid

## Abstract

Background

The study aims to assess maternal and fetal outcomes in pregnancy complicated by a fibroid uterus.

Materials and methods

Sixty-four adult women patients with fibroids in age range of 25-45 years were selected. Fetal and maternal outcomes were recorded.

Results

Thirty-four patients were in the age group of 25-35 years, and 50 were aged 35-45 years of age. Primigravida was seen in 52 and multigravida in 32. The common types were intramural in 40, submucosal in 16, pedunculated in seven, and subserosal in 21 cases. Size >5 cm was seen in 38 and 2 cm-5 cm in 46 patients. Common symptoms were preterm labor pain in 50, abdominal pain in 12, and urinary tract infection (UTI) in 22 cases. The common sites were the anterior wall fibroid in 42, posterior wall in 22, and lateral wall in 20 cases. Complications reported in our study were premature rupture of membranes (PROMs) in 12, threatened preterm in 11, associated infertility in five, intrauterine growth retardation (IUGR) in two, malpresentation in seven, postpartum hemorrhage (PPH) in four, preterm labor in six, and abruption in eight cases. The difference was observed to be significant (P<0.05).

Conclusion

Common complications were PROM, threatened preterm, associated infertility, IUGR, malpresentation, PPH, preterm labor, and abruption.

## Introduction

Uterus encounters multiple tumors, of which leiomyomas are the most common. It is benign in nature, involving smooth muscle cells [1]. It is not encapsulated and includes about 40-60% of the population [2]. There is about a 1.2-10.7% prevalence rate of leiomyomas affecting pregnant women. They are usually not symptomatic in pregnancy [3]. These are diagnosed accidentally when women undergo routine ultrasound examinations (USGs) during trimesters. Sometimes, they are not detected on USG owing to thick myometrium [4].

Uneventful pregnancies have been noted in females with small fibroids. Some of them may show some complications. Pregnancy outcomes are affected by factors related to fibroids, such as number, shape, size, and site [5]. These women may show nausea, vomiting, pyrexia, and hemorrhagic infarction, which are evident at 20-22 weeks of pregnancy. The chances of miscarriage cannot be overruled in the case of intramural or submucosal fibroids. A decreased rate of pregnancy and implantation failure has been seen in females with fibroids [6]. The rate is quite common with submucosal fibroids. Patients present with moderate abdominal colic in response to fibroid red degeneration, non-sessile fibroids resulting in a torsion. Myomectomy is highly indicated in early pregnancy, even in the case of strict contraindication. This is highly significant for intractable and recurrent pain [7]. Symptoms of urgency and increased frequency and urinary tract infections (UTIs) are seen in the mother. Neonatal complications such as low birth weight babies secondary to fetal growth restriction with no perinatal morbidity, mortality, and congenital anomalies are common [8,9]. Considering this, we attempted the present study with the aim of assessing maternal and fetal outcomes in pregnancy complicated by a fibroid uterus.

## Materials and methods

A total of sixty-four adult women patients with fibroids in the age range of 25-45 years were selected. All selected women gave their written consent for participation. Ethical approval was received from the institutional committee reviewing ethical issues and the “Institutional Ethical Committee (IEC) number is “HH/2021/12-0991.”

The demographic data of each patient was recorded. Parameters such as maternal age, parity, socioeconomic status, and educational status were recorded. A history of family, infertility and previous abortions was obtained. The history of antepartum hemorrhage (APH), size, symptoms, and site and type of fibroids were recorded. The fetal parameters, such as birth weight, term or preterm, and neonatal intensive care units (NICUs) admissions, were recorded. The fetal parameters were recorded by a trained pediatrician and appearance, pulse, grimace, activity, and respiration (APGAR) scoring was done at one and five minutes. The results of the present study were compiled and entered into an MS Excel sheet (Microsoft Corporation, Redmond, Washington, USA) for statistical analysis. The level of significance was set at below 0.05.

## Results

Our study had 34 patients in the age group of 25-35 years and 50 patients aged 35-45 years of age. A significant difference was found (P<0.05) (Table 1).

**Table 1:** Distribution of age.

Age group	Number	P-value
25-35 years	34	<0.05
35-45 years	50	
Total	84	

Our study showed that primigravida was seen in 52 and multigravida in 32. A significant difference was found (P<0.05) (Table 2).

**Table 2:** Distribution based on gravida.

Gravida	Number	P-value
Primigravida	52	<0.05
Multigravida	32	
Total	84	

Our study showed that the common type was intramural in 40, submucosal in 16, pedunculated in 7, and subserosal in 21 cases. Size >5 cm was observed in 38 patients, and 2 cm-5 cm was observed in 46 patients. Common symptoms were preterm labor pain in 50, abdominal pain in 12, and urinary tract infection (UTI) in 22 cases. The common sites were the anterior wall fibroid in 42, posterior wall in 22, and lateral wall in 20 cases. A significant difference was found (P<0.05) (Table 3).

**Table 3:** Assessment of parameters. UTI: urinary tract infection.

Parameters	Variables	Number	P-value
Type	Intramural	40	<0.05
	Submucosal	16	
	Pedunculated	7	
	Subserosal	21	
Size	>5 cm	38	<0.05
	2 cm-5 cm	46	
Symptoms	Preterm labor pain	50	<0.05
	Pain abdomen	12	
	UTI	22	
Site	Anterior wall fibroid	42	<0.05
	Posterior wall	22	
	Lateral wall	20	

Complications reported in our study were premature rupture of membranes (PROMs) in 12, threatened preterm in 11, associated infertility in five, intra-uterine growth retardation (IUGR) in two, malpresentation in seven, postpartum hemorrhage (PPH) in four, preterm labor in six, and abruption in eight cases. The difference was significant (P<0.05) (Table 4).

**Table 4:** Complications. PROM: premature rupture of membranes; IUGR: intra-uterine growth retardation; PPH: postpartum hemorrhage.

Complications	Number	P-value
PROM	12	<0.05
Threatened preterm	11	
Associated infertility	5	
IUGR	2	
Malpresentation	7	
PPH	4	
Preterm labor	6	
Abruption	8	

## Discussion

Myomas are considered to be the most commonly found benign smooth muscle tumors of the uterus. It is seen in 35-77% of females of reproductive age [10,11]. Menstrual disorders and pelvic pain are common in these patients [12]. They have a great impact on fertile potential and the outcome of pregnancy. The occurrence of fibroids is recorded in 0.1-10.5% of all pregnant cases. It is evident that as the age of the mother increases, the chances of fibroids increase [13,14]. They are also common among nulliparas [15]. The dangers of cesarean delivery, presentation of breech, malposition, and premature delivery are quite common in pregnancy with myoma [16]. It is observed that a fibroid <5 cm has the potential to breed in pregnancy. With the increase in fibroid size, there is a significant increase in the size of the fibroid [17,18]. In the present study, maternal as well as fetal outcomes were determined in pregnancy complicated by a fibroid uterus.

Our study showed that 34 patients were seen in the age group 25-35 years and 50 in 35-45 years of age. Bhat et al. [19] in their study conducted on 5,043 deliveries had 30 cases of fibroid during pregnancy. Results showed that 50% of cases were seen in the second decade of life, out of which 18 (60%) were primigravida. There were 26 (86.66%) cases of lower segment cesarean section (LSCS), premature vaginal delivery occurred in 4 (13.33%), 16.66% showed malpresentation, 15% underwent myomectomy, and 20% showed premature rupture of the membrane. It showed that in 16.6% (5) infertility was treated. Symptoms found were abdominal pain in 20 (66.66%), postpartum hemorrhage, placenta abruptio, IUGR, and low birth weight in 3 (10%), 3.3%, 16%, and 26.66%, respectively.

We observed that primigravida was seen in 52 and multigravida in 32. The common types were intramural in 40, submucosal in 16, pedunculated in 7, and subserosal in 21 cases. Size >5 cm was observed in 38 patients, and 2 cm-5 cm was observed in 46 patients. Common symptoms were preterm labor pain in 50, abdominal pain in 12, and UTI in 22 cases. The common site were the anterior wall fibroid in 42, posterior wall in 22, and lateral wall in 20 cases. Poovathi et al. [20] conducted a study on 30 women. 22 (73.3%) cases were seen in multigravidae and 8 (26.6%) in primigravidae. Fibroids in pregnancy range from 0.01% to 10.7%. Symptoms were seen in 10 (33.3%) females during pregnancy. 10 (33.3%) were known cases of fibroid becoming pregnant, the rest 20 (66.6%) were diagnosed as having fibroid during antenatal visits. Results showed that 23.3% (7) patients had pain, 13.3% (4) had threatened preterm labor, 10% (3) had a spontaneous miscarriage, 10% (3) had anemia, and 10% of patients showed placenta previa. Complete gestation was seen among 27 women (90%), of which normal vaginal delivery occurred in 8 (29.6%) women, outlet forceps were applied in one woman, and ventouse in one woman. 59.2% (16) patients underwent lower segment cesarean section and one had a cesarean hysterectomy.

Complications reported in our study were PROM in 12, threatened preterm in 11, associated infertility in five, IUGR in two, malpresentation in seven, PPH in four, preterm labor in six, and abruption in eight cases. The limitation of the present study is the small patient count and short follow-up.

## Conclusions

The study was conducted extensively on the types of patients being admitted to a tertiary care center. The results of the study reveal that common complications arising due to a fibroid uterus were PROM, threatened preterm, associated infertility, IUGR, malpresentation, PPH, preterm labor, and abruption. The study advocates to be cautious whenever a pregnant patient together with a fibroid uterus comes to visit a maternal-fetal well-being clinic. Keeping in mind the probable common complications, one can manage the maternal and child's health in the best possible way.
